# The Clinical Spectrum of Cutaneous Tuberculosis: A Case Series Emphasizing Prompt Recognition and Treatment

**DOI:** 10.7759/cureus.86906

**Published:** 2025-06-28

**Authors:** Pavithra C, Jince Ann Jose, Ghanshyam Verma, Lakshmi Priya V, Elen Abraham

**Affiliations:** 1 Pulmonary Medicine, Sree Balaji Medical College and Hospital, Chennai, IND; 2 Respiratory Medicine, Sree Balaji Medical College and Hospital, Chennai, IND; 3 Pathology, Sree Balaji Medical College and Hospital, Chennai, IND

**Keywords:** cutaneous tuberculosis, extrapulmonary tuberculosis, lupus vulgaris, scrofuloderma, tuberculosis gumma

## Abstract

Cutaneous tuberculosis (CTB) represents a minor proportion of all cases of extrapulmonary tuberculosis (EPTB), with lupus vulgaris (LV) and cervical scrofuloderma (CS) being the most prevalent forms. Metastatic tuberculosis abscesses (MTBA), tuberculids (TBDs), and tuberculosis verrucosa cutis (TBVC) are less common variations. Since CTB frequently occurs in individuals with strong immune systems who have experienced a hypersensitivity reaction to an extracutaneous source of *Mycobacterium tuberculosis* (MTB), it can be challenging to diagnose. This case series emphasizes the diverse clinical manifestations of CTB, highlighting the urgent need for prompt diagnosis and effective treatment.

## Introduction

Cutaneous tuberculosis (CTB) accounts for approximately 1-1.5% of all cases of extrapulmonary tuberculosis (EPTB) [1,2]. The primary causative organism is *Mycobacterium tuberculosis* (MTB), although *Mycobacterium bovis* and the bacillus Calmette-Guérin (BCG) strain have also been implicated in rare instances. The clinical manifestations of CTB are highly variable and are significantly influenced by the anatomical site of infection and the host’s immune response [1,2].

Historically referred to as the “king’s evil”, a term based on the belief that royal touch could heal the afflicted, forms such as scrofuloderma (SFD) and lupus vulgaris (LV) are among the earliest described variants of CTB. The term “lupus,” meaning “wolf” in Latin, was used to denote ulcerative lesions that mimicked wolf bites [3].

During the 19th and early 20th centuries, CTB represented a significant public health burden. However, its prevalence markedly declined with improvements in hygiene, living standards, widespread BCG vaccination, and the introduction of effective anti-tubercular treatment (ATT). Despite these advancements, the disease has re-emerged in recent decades, largely due to increased usage of immunosuppressive therapies, the rise of multidrug-resistant tuberculosis (MDR-TB), and the global prevalence of human immunodeficiency virus (HIV) infection [3].

Globally, CTB remains a diagnostic challenge due to its diverse clinical presentation. The condition includes both multibacillary forms, such as SFD and tuberculous chancre (TC), and paucibacillary forms, such as LV and tuberculosis verrucosa cutis (TBVC). Accurate diagnosis often necessitates a multidisciplinary approach involving clinical evaluation, histopathological analysis, and microbiological confirmation. Early diagnosis and prompt initiation of appropriate ATT are vital for optimal outcomes and for preventing long-term complications [4-6]. This case series aims to educate clinicians on the varied presentations of CTB, thereby enhancing clinical suspicion, diagnostic accuracy, and management strategies for this uncommon but important manifestation of tuberculosis (TB).

## Case presentation

Case 1

**Figure 1:**
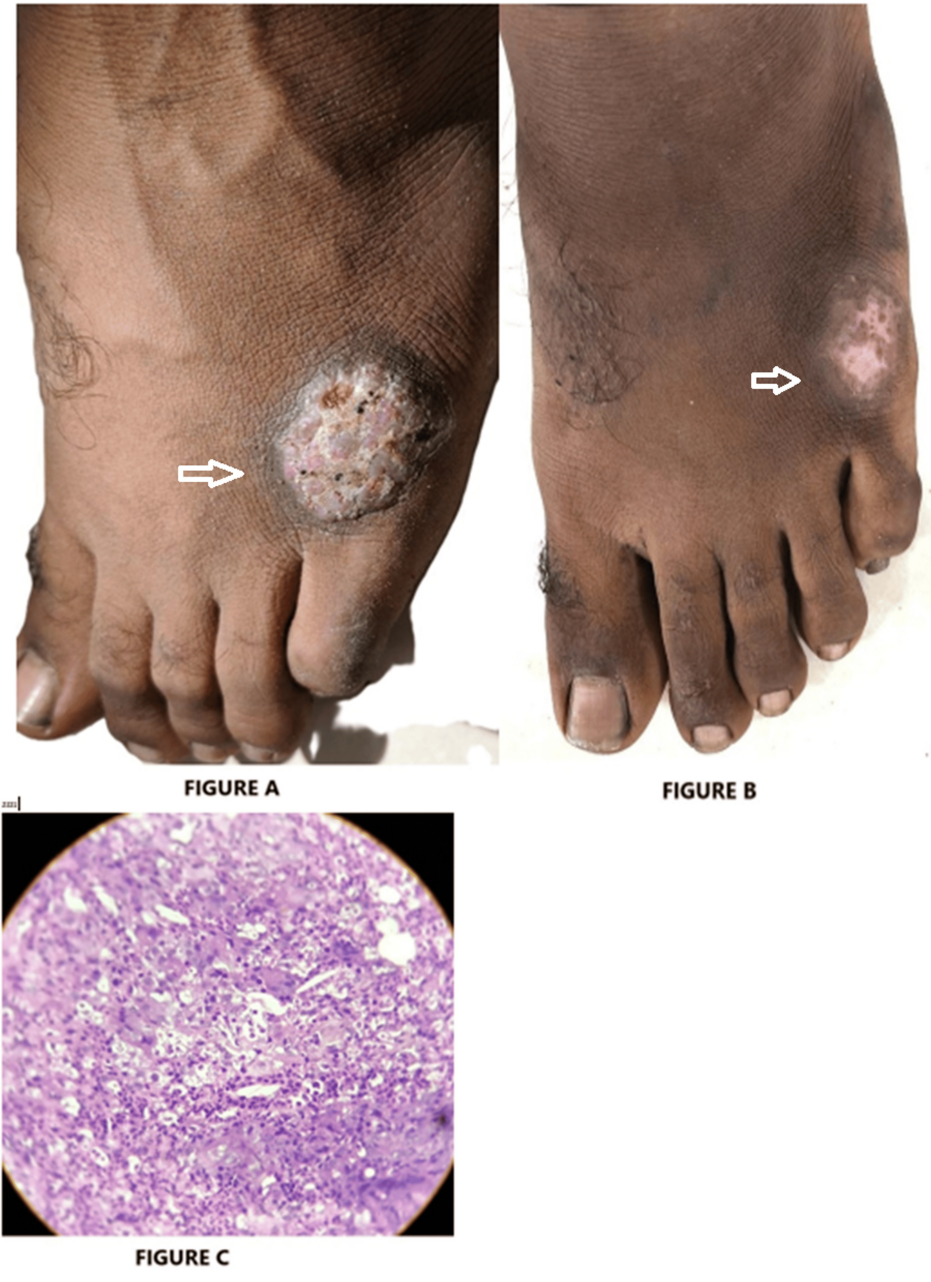
TBVC Lesion: (A) clinical presentation before treatment; (B) post-treatment appearance; (C) histopathological image of TBVC showing papillomatosis, mononuclear cell inflammation, and epithelioid granuloma.

A 24-year-old male software developer presented with a solitary red plaque over the lateral aspect of his left foot, over the fifth metatarsal region. The lesion had been slowly increasing in size over the past three years. The patient denied any respiratory or other constitutional symptoms. There was no personal or family history of TB or other chronic illnesses. On local examination, a 2.5 × 2.5 cm well-defined, non-tender, erythematous plaque was noted over the dorsolateral aspect of the left foot. Routine hematological and biochemical investigations were within normal limits. A Mantoux test (tuberculin skin test, TST) revealed a strongly positive reaction, with 21 mm of induration at 72 hours. A punch biopsy of the lesion was performed. Histopathological examination reported findings consistent with TBVC. The patient was initiated on first-line ATT consisting of isoniazid, rifampicin, pyrazinamide, and ethambutol, according to weight band. The patient was monitored for compliance and adverse effects throughout the treatment. Within six weeks of initiating ATT, the lesion showed significant regression in size and inflammation, eventually healing with residual atrophic scarring (Figure 1A-C).

Case 2

**Figure 2:**
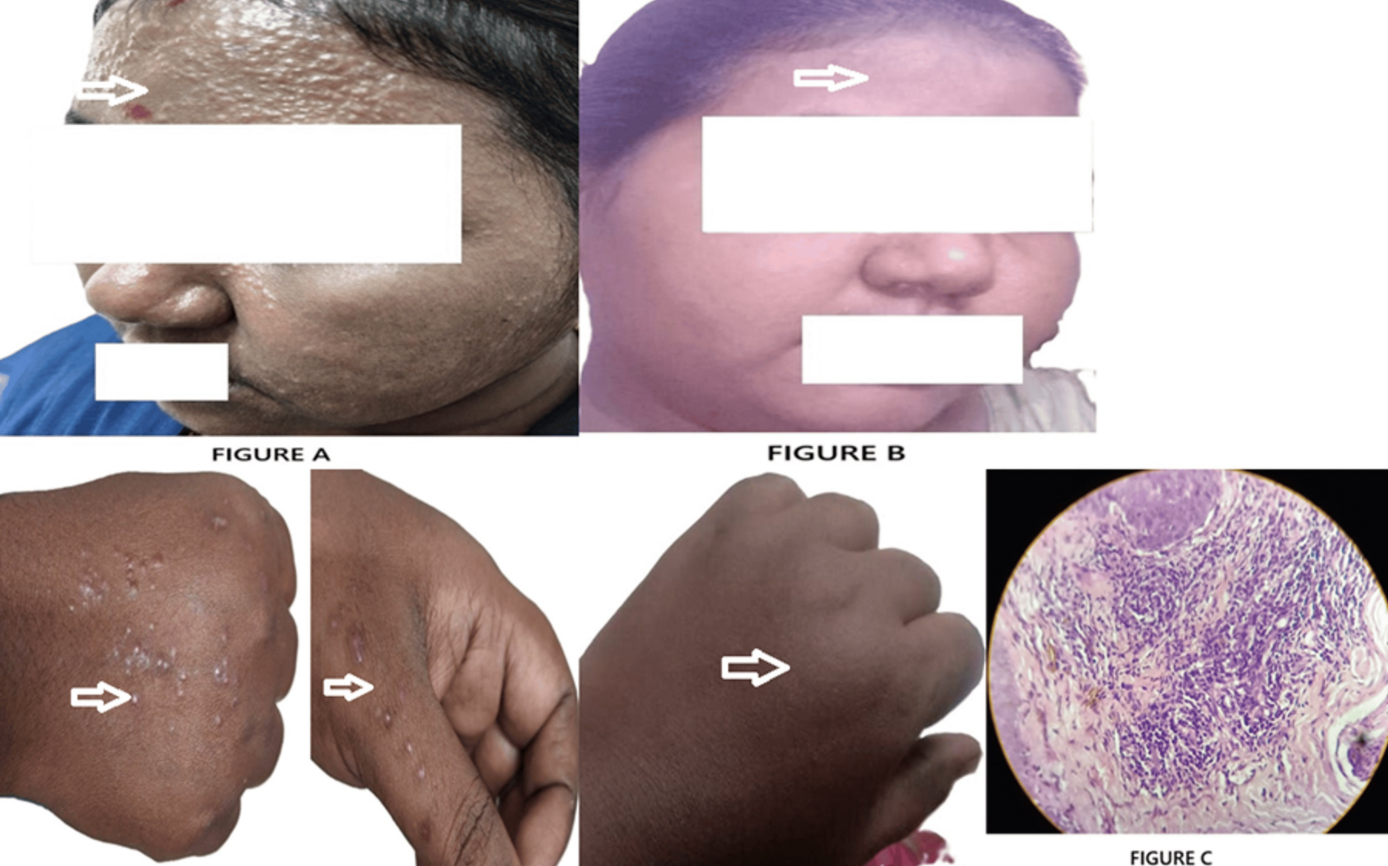
Lichen scrofulosorum (LS): (A) clinical presentation before treatment; (B) post-treatment appearance; (C) histopathological image of LS epithelioid granuloma with Langhans type of giant cell.

A 35-year-old female homemaker presented with a five-year history of multiple mildly pruritic papular eruptions over her face and back. She had been treated intermittently with topical and systemic corticosteroids, antibiotics, and antihistamines, experiencing only temporary relief with frequent relapses. The patient reported no systemic symptoms. Her medical and family history was non-contributory, with no known history of TB. On local examination, multiple discrete, erythematous papules were observed over the face and dorsum of the hands. There was no ulceration, scaling, or scarring. The rest of the physical examination was unremarkable. Routine blood investigations were within normal limits. The TST was negative. A skin biopsy was performed from a representative lesion. Histopathological examination revealed findings characteristic of lichen scrofulosorum (LS). A diagnosis of LS was made based on clinical and histopathological correlation. The patient was started on ATT, according to weight band. The patient tolerated the treatment well, without any reported side effects. Significant clinical improvement was noted within four weeks, with complete resolution of lesions by the end of two months. The patient has been followed up regularly for 18 months, with no recurrence of lesions or new complaints (Figure 2A-C).

Case 3

**Figure 3:**
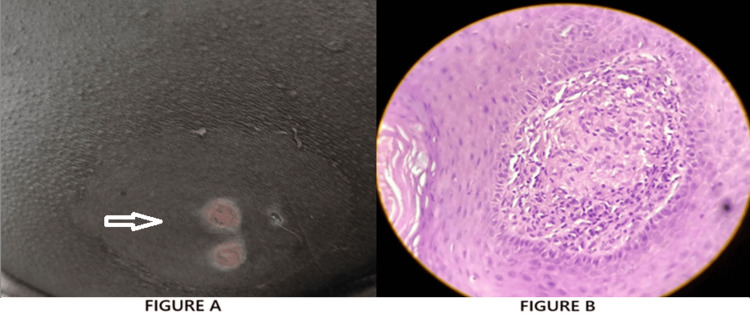
Tuberculous gumma (TG): (A) clinical presentation; (B) histopathological image of TG showing multinucleated giant cell in dermis.

A 32-year-old man presented with multiple painful swellings over the left gluteal region of one-month duration. The lesions initially responded partially to antibiotics after pus culture revealed Enterococcus faecalis, but recurred shortly after cessation of treatment. There was no history of trauma, systemic symptoms such as fever or weight loss, or any known contact with TB. The patient had no significant personal or family history of TB or immunosuppressive disorders. On physical examination, multiple clustered, tender abscesses were noted over the left gluteal region, without regional lymphadenopathy. No similar lesions were found elsewhere. A differential diagnosis of pyemic abscesses, hidradenitis suppurativa, and atypical infections was considered. Routine hematological and biochemical investigations were within normal limits. A TST was strongly positive, measuring 25 mm of induration at 72 hours. A punch biopsy of the lesion was performed. Acid-fast bacilli were identified on Ziehl-Neelsen staining of pus samples. These findings were consistent with a diagnosis of tuberculous gumma (TG). The patient was initiated on weight-based ATT. The response to treatment was excellent, with marked reduction in pain and inflammation within three weeks. Complete healing of all lesions occurred by the end of two months, leaving minimal scarring. The patient tolerated therapy well, with no reported side effects (Figure 3A-C).

Case 4

**Figure 4:**
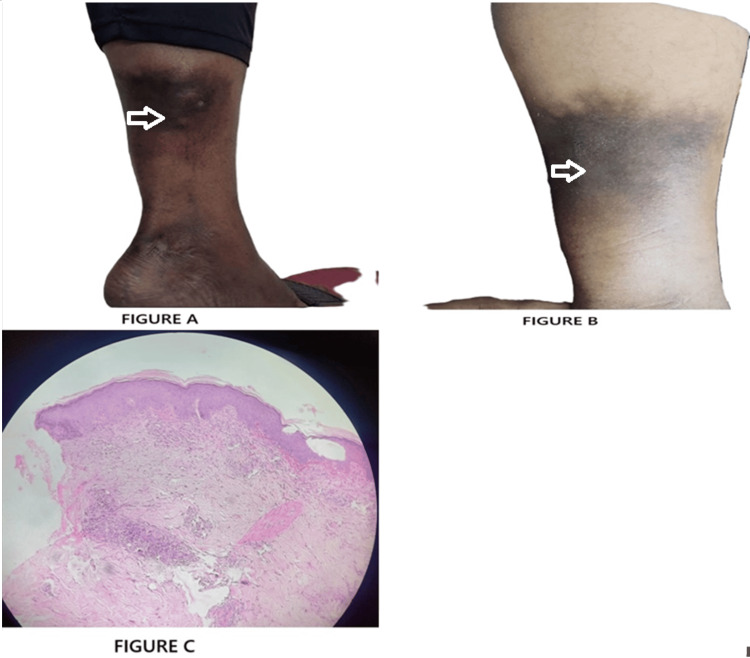
Erythema induratum (EI): (A) Lesion prior to therapy; (B) resolution following treatment; (C) histopathological section of EI lobular panniculitis, perivascular vasculopathy reaction.

A 43-year-old male presented with persistent papule-nodule-like lesions over the left leg for the past two months. The lesions were mildly tender, firm, and non-ulcerated. There were no systemic symptoms or prior history of TB or TB exposure. The patient's medical and family history was unremarkable. On local examination, indurated erythematous nodules were noted on the posterior aspect of the left lower leg. No ulceration or discharge was present. A punch biopsy of one lesion was performed. Histopathological examination revealed findings consistent with erythema induratum (EI). An interferon-gamma release assay (IGRA) was performed, yielding a positive result (0.781 IU/mL; positive ≥ 0.35 IU/mL). Based on the clinical presentation, histopathological findings, and positive IGRA, a diagnosis of EI (Bazin’s disease) associated with latent TB was established. The patient was started on standard weight-based ATT. Gradual but significant clinical improvement was observed over the following weeks. The nodules decreased in size and tenderness, with near-complete resolution by the end of two months. The patient tolerated the treatment well, with no reported side effects (Figure 4A-C).

Case 5

**Figure 5:**
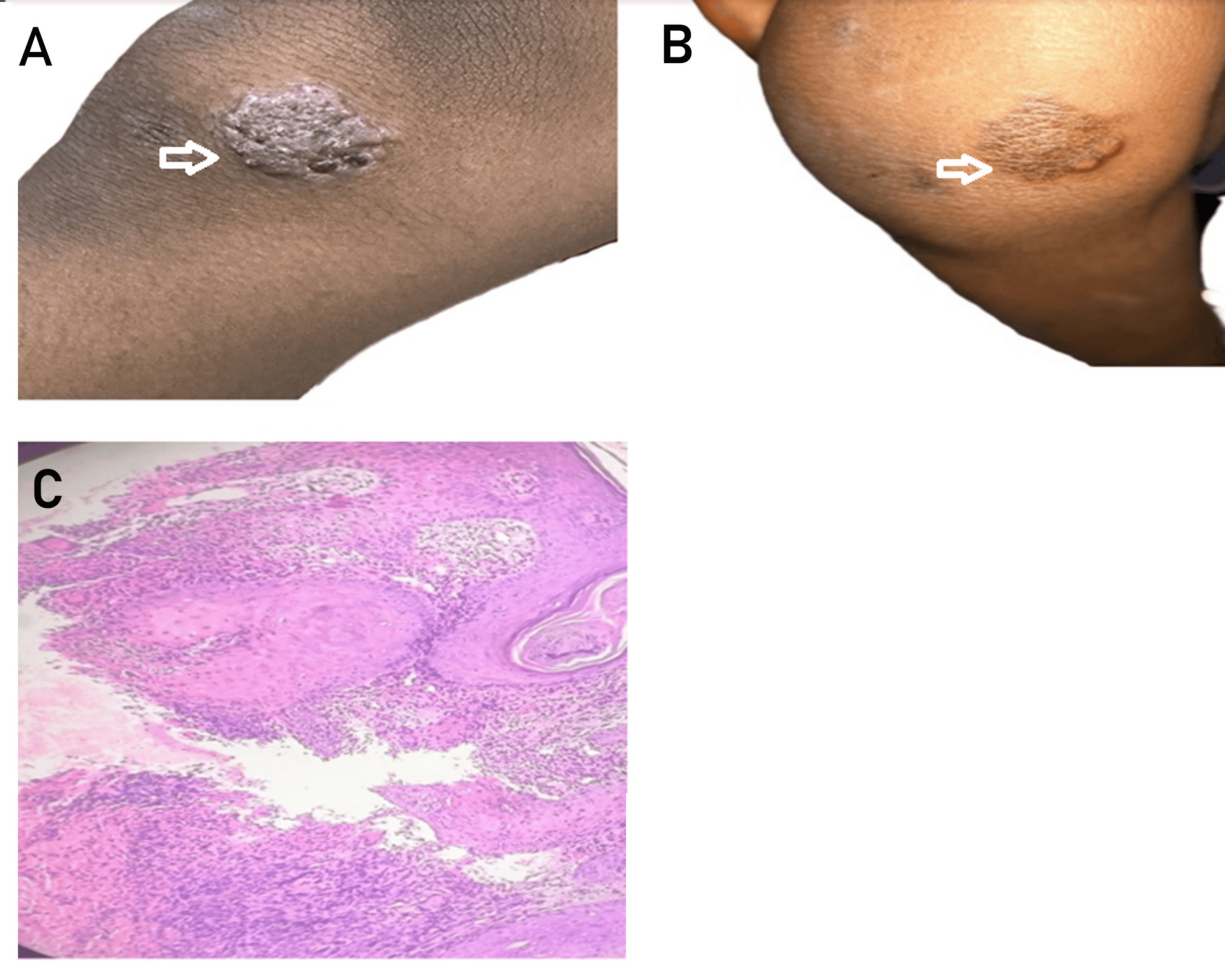
Lupus vulgaris (LV): (A) clinical presentation before treatment; (B) post-treatment appearance; (C) histopathological image of LV showing ill defined granuloma with inflammatory cells and Langhans type of giant cells.

A five-year-old girl presented with a six-month history of a reddish-brown nodular lesion over the right knee. The lesion had gradually increased in size but remained asymptomatic. There was no history of trauma or constitutional symptoms. On examination, a well-defined, firm, reddish-brown nodular lesion was noted on the extensor surface of the right knee. The surface was dry, slightly scaly, and non-tender. A punch biopsy of the lesion was performed. Histopathological examination revealed features consistent with a diagnosis of lupus vulgaris (LV). Routine blood investigations were within normal limits. Based on the clinical presentation and histopathological findings, showing ill-defined granuloma with inflammatory cells and Langhans-type giant cells, the diagnosis was established. The patient was started on ATT. Regular follow-up showed progressive resolution of the lesion (Figure 5A-C).

## Discussion

CTB is an uncommon extrapulmonary manifestation of MTB, accounting for only 1-1.5% of all EPTB cases [7]. It presents a diagnostic challenge due to its varied clinical morphology and resemblance to numerous dermatological conditions. This case series highlights five distinct CTB variants, each demonstrating unique clinicopathological features that underscore the importance of integrative diagnostic approaches (Table 1).

**Table 1:** comparative summary table of CTB cases, capturing key clinical, diagnostic, and treatment outcome related details CTB: cutaneous tuberculosis, TBVC: tuberculosis verrucosa cutis, LS: lichen scrofulosorum, TG: tuberculous gumma, EI: erythema induratum, LV: lupus vulgaris, TST: tuberculin skin test, ATT: antitubercular therapy, HRZE: isoniazid, rifampicin, pyrazinamide, and ethambutol, IGRA: interferon gamma release assay.

Case	Age/Sex	Clinical Features	Histopathological Findings	Diagnostic Tests	Treatment	Outcome
1. TBVC	24/M	Solitary, erythematous, well-defined plaque over dorsolateral left foot (5th metatarsal), slowly progressing over 3 years	Papillomatosis, mononuclear cell infiltration, epithelioid granuloma	TST: 21 mm; Routine labs: normal; Biopsy: diagnostic	First-line ATT (HRZE), weight-based	Significant regression by 6 weeks; healed with atrophic scarring
2. LS	35/F	Multiple mildly pruritic, erythematous papules on face and back; relapsing.	Granulomatous inflammation with Langhans-type giant cells	TST: Negative; Biopsy: diagnostic for LS; Routine labs: normal	First-line ATT, weight-based	Complete resolution in 2 months; no recurrence at 18-month follow-up
3. TG	32/M	Multiple painful, tender, recurrent gluteal abscesses.	Multinucleated giant cells in the dermis	TST: 25 mm; Biopsy: diagnostic	First-line ATT, weight-based	Rapid improvement in 3 weeks; complete healing by 2 months with minimal scarring
4. EI	43/M	Mildly tender, indurated nodules over the posterior left leg; no ulceration or discharge	Lobular panniculitis	IGRA: Positive (0.781 IU/mL); Biopsy: diagnostic	First-line ATT, weight-based	Gradual regression; near-complete resolution by 2 months
5. LV	5/F	Firm, reddish-brown nodular lesion over right knee; dry, slightly scaly, non-tender	Granulomatous infiltration with Langhans-type giant cells	TST: negative; Biopsy: diagnostic for LV; Routine labs: normal	First-line ATT, weight-based	Progressive resolution on follow-up by the end of 2 months with scaring

Case 1 demonstrated a classical presentation of TBVC, a paucibacillary form resulting from exogenous reinfection in a sensitized individual. Despite negative AFB staining and GeneXpert MTB/RIF assay, the diagnosis was substantiated by a strongly positive TST and characteristic biopsy findings. The lesion’s resolution with standard ATT further affirmed the diagnosis. This aligns with findings by Roelan et al. [7], who highlighted the diagnostic limitations in paucibacillary forms and the need for histopathological correlation.

Case 2, a prototypical TBD, presented with recurrent papules and no systemic involvement. Although the TST and imaging were negative, histological analysis revealed granulomatous inflammation with Langhans-type giant cells, establishing the diagnosis. Bravo and Gotuzzo [1] described such TBDs as manifestations of strong immune reactions in immunocompetent hosts (ICH), often with negative bacteriological test patterns mirrored in this case.

Case 3, a multibacillary form, presented with recurrent gluteal abscesses initially misdiagnosed as bacterial infections. Histopathology showed necrotizing granulomas and AFB positivity. The patient’s rapid response to ATT following antibiotic failure highlights the clinical mimicry of CTB, a diagnostic pitfall echoed in the literature. Nguyen et al. [8] reported similar diagnostic dilemmas, emphasizing the need for heightened suspicion and biopsy in atypical or non-healing abscesses (ANHA).

Case 4, diagnosed as EI, was supported by histopathological evidence of lobular panniculitis and a positive IGRA. EI is a deep tuberculid linked to latent tuberculosis infection (LTBI). Such vasculitic lesions often lack direct microbiological evidence, and diagnosis rests on clinicopathological features, as noted by Bravo et al. [1] and Supekar et al. [9]. The role of IGRA as a supportive diagnostic tool, especially in deep CTB lesions, is well documented in these studies.

Case 5, a pediatric case of LV, was confirmed via biopsy showing Langhans-type giant cells and granulomatous infiltration. Despite negative AFB results, classical clinical and histological features sufficed for diagnosis. Supekar et al. [9] identified LV as the most prevalent adult CTB form in India, though pediatric involvement is not uncommon, especially in TB-endemic regions (TERs).

This series corroborates the diagnostic complexity of CTB described by Roelan et al. [7] and Bravo et al. [1], particularly the diagnostic reliance on clinicopathological correlation in paucibacillary and TBD forms. Furthermore, our experience resonates with Nguyen et al. [8], who stressed the mimicry of multibacillary CTB lesions with common bacterial infections, as seen in our case of TG.

While conventional diagnostics such as AFB staining, histology, and IGRA remain mainstays, newer tools like metagenomic next-generation sequencing (mNGS) offer increased sensitivity. Kong et al. [10] demonstrated the efficacy of mNGS in detecting mycobacterial DNA even in smear-negative samples, which may prove beneficial in future similar cases, particularly when standard diagnostics are inconclusive.

CTB’s mimicry of other dermatoses, including mycetoma, hidradenitis suppurativa (HS), and vasculitic panniculitis (VP), is a well-acknowledged concern. Ahmed et al. [11] presented a CTB case simulating mycetoma, while our series included lesions resembling bacterial abscesses and vasculitis. Such deceptive presentations further emphasize the need for a thorough diagnostic workup, including biopsy and TB-specific tests.

The pathophysiology of CTB often involves a delayed-type hypersensitivity (DTH) response to MTB antigens, particularly in ICH. This immunological containment results in paucibacillary lesions that are AFB-negative, as seen in TBVC and LS. Strong cell-mediated immune (CMI) responses localize but fail to eradicate the pathogen, resulting in chronic lesions that may be mistaken for non-infectious dermatoses [1,8].

Early diagnosis and treatment initiation are critical. Standard ATT remains the cornerstone of management and yields excellent results when started promptly. However, delays can lead to complications such as chronic ulceration, scarring, and secondary bacterial infections (SBI) [11]. In TERs, clinicians must maintain a high index of suspicion for CTB in chronic, non-resolving skin lesions, especially when routine therapy fails.

## Conclusions

CTB, although constituting a minor fraction of EPTB cases, poses significant diagnostic and therapeutic challenges due to its diverse clinical presentations and frequent overlap with other dermatological conditions. This case series illustrates the clinical heterogeneity of CTB through five cases, each exemplifying a distinct morphological variant: TBVC, LS, TG, EI, and LV. These incidents highlight the importance of maintaining a high level of suspicion, particularly in areas where the condition is endemic or while assessing chronic, non-healing, or treatment-resistant skin lesions. Traditional diagnostic tools such as the TST, histopathological examination, AFB staining, and IGRA play a crucial role in confirming the diagnosis. In paucibacillary forms, where microbiological evidence may be lacking, clinicopathological correlation becomes pivotal.

Importantly, all five patients in this series demonstrated favorable responses to first-line ATT, highlighting the effectiveness of timely pharmacological intervention. Early recognition and appropriate treatment not only lead to complete clinical resolution but also prevent long-term sequelae such as scarring, disfigurement, and dissemination. Broader screening procedures, with particular focus on pediatric and unusual cases, should be taken into consideration in endemic areas in order to decrease missed or delayed diagnoses. Public health surveillance can be strengthened, and dermatological examination incorporated into TB control programs, efforts to greatly enhance early detection and results. Multicentric studies are also required to describe CTB more accurately, track resistance trends, and develop evidence-based treatment durations for certain subtypes.

## References

[1] Bravo FG, Gotuzzo E (2007). Cutaneous tuberculosis. Clin Dermatol.

[2] Santos JB, Figueiredo AR, Ferraz CE, Oliveira MH, Silva PG, Medeiros VL (2014). Cutaneous tuberculosis: epidemiologic, etiopathogenic and clinical aspects - part I. An Bras Dermatol.

[3] Khadka P, Koirala S, Thapaliya J (2018). Cutaneous tuberculosis: clinicopathologic arrays and diagnostic challenges. Dermatol Res Pract.

[4] Frankel A, Penrose C, Emer J (2009). Cutaneous tuberculosis: a practical case report and review for the dermatologist. J Clin Aesthet Dermatol.

[5] Barbagallo J, Tager P, Ingleton R, Hirsch RJ, Weinberg JM (2002). Cutaneous tuberculosis: diagnosis and treatment. Am J Clin Dermatol.

[6] Zeggwagh Z, Azendour H, Znati K, Senouci K (2023). Atypical cutaneous tuberculosis with an unusual course. Int J Mycobacteriol.

[7] Roelan T (2021). Practical review of diagnosis and management of cutaneous tuberculosis in Indonesia. Eur J Med Health Sci.

[8] Nguyen KH, Alcantara CA, Glassman I (2023). Cutaneous manifestations of Mycobacterium tuberculosis: a literature review. Pathogens.

[9] Supekar BB, Wankhade VH, Singh RP, Ghanate TD, Bhat D (2021). Clinical spectrum of cutaneous tuberculosis in Central India: a retrospective study. Indian Dermatol Online J.

[10] Kong M, Li W, Kong Q, Dong H, Han A, Jiang L (2022). Application of metagenomic next-generation sequencing in cutaneous tuberculosis. Front Cell Infect Microbiol.

[11] Ahmed A, Hagelnur AA, Eltigani HF, Siddig EE (2023). Cutaneous tuberculosis of the foot clinically mimicking mycetoma: a case report. Clin Case Rep.

